# Enhancing Knee Arthrocentesis: Boosting Operator Confidence With Point-of-Care Ultrasound (POCUS)

**DOI:** 10.7759/cureus.111317

**Published:** 2026-06-22

**Authors:** Mehak Sharma, Carolina E Valdes-Guicciardi, Aditi Kumar, Guillermo Izquierdo-Pretel

**Affiliations:** 1 Internal Medicine, Florida International University, Herbert Wertheim College of Medicine, Miami, USA; 2 Family Medicine, Penn State College of Medicine, Hershey, USA; 3 Hospital Medicine, Jackson Memorial Hospital, Miami, USA

**Keywords:** arthrocentesis, gout, point-of-care ultrasound, procedural training, resident education

## Abstract

Point-of-care ultrasound (POCUS) is increasingly recognized as a valuable adjunct for musculoskeletal procedures, enhancing both diagnostic accuracy and procedural success. We report the case of a 34-year-old man with gout presenting with acute polyarthralgia and left knee effusion. A postgraduate year one (PGY-1) internal medicine resident performed his first arthrocentesis under POCUS guidance, achieving first-attempt success. Synovial fluid analysis revealed elevated uric acid, and urate-lowering therapy was initiated, with rapid symptom resolution. This case illustrates how POCUS provides real-time visualization that improves accuracy, reduces procedural attempts, and bolsters novice operator confidence. Incorporating POCUS-guided arthrocentesis into residency training may enhance procedural proficiency, expedite diagnosis, and improve patient outcomes.

## Introduction

Point-of-care ultrasound (POCUS) can aid in the diagnosis and management of joint effusion. While anatomical landmarks have traditionally been used to guide arthrocentesis, ultrasound-guided aspirations yield an overall higher success rate (89%) compared to landmark use alone (58%) [1]. POCUS is a form of diagnostic ultrasonography that uses high-frequency sound waves emitted from a transducer, which travel through tissue and are reflected back at interfaces with different acoustic properties. These returning echoes are processed in real time to generate diagnostic images, allowing visualization of anatomic structures and fluid collections at the bedside [1,2]. POCUS provides real-time visualization and localization of joint effusions, which can help improve operator confidence and increase procedural success among novice healthcare providers. Traditional landmark-guided arthrocentesis relies on the palpation of surface anatomy and is performed without direct visualization of the joint space. However, this approach can be challenging in patients with small effusions, anatomic variants, or significant soft tissue swelling, potentially resulting in multiple needle passes, incomplete aspiration, inadvertent contact with cartilage or bone, and increased patient discomfort [1,3]. Ultrasound guidance addresses these limitations by allowing real-time visualization of the effusion, surrounding structures, and needle placement, thereby facilitating more accurate needle placement and fluid aspiration [1,3]. A randomized, double-blind, controlled study demonstrated that ultrasound-guided corticosteroid injections were 68% more accurate than those performed using anatomical landmarks [4]. Greater injection accuracy was associated with significant improvements in joint function by increasing joint mobility [4]. This finding underscores the value of ultrasound guidance for all physicians, as it enhances efficiency and leads to better long-term patient outcomes.

Recognizing these advantages, we present this case to highlight not only the clinical utility of POCUS in knee arthrocentesis but also its role in fostering procedural confidence for novice operators. Our main proposal is that structured integration of POCUS-guided arthrocentesis into residency training can enhance technical proficiency, reduce procedural attempts, and ultimately improve patient care outcomes. In high-stakes cases, such as the diagnosis and treatment of septic arthritis, this approach could prove critical in optimizing timely and accurate interventions [5].

## Case presentation

A 34-year-old Black male with a history of gout presented with acute-onset joint pain and swelling of the left knee. Symptoms began on the lateral aspect of his left foot two weeks earlier and progressively involved both feet, ankles, and the left knee. The pain did not improve with ibuprofen or warm compresses. His mobility was significantly limited, requiring the use of crutches and a wheelchair. He denied fever, chills, nausea, vomiting, chest pain, abdominal pain, diarrhea, shortness of breath, sick contacts, travel to forested areas, tick bites, or recent sexual activity. Diagnosed with gout in 2018, he reported similar flares up to four times per year. He had never undergone arthrocentesis and stated that he was unsure whether he had ever been started on medication for gout; however, he was not using any medications at the time of admission.

On admission, he was hemodynamically stable, alert, and in no acute distress. Examination revealed trace left lower extremity edema and a left knee effusion, with mild tenderness and erythema over the left knee. Laboratory tests showed uric acid of 9.6 mg/dL (reference range: 3.4-7.0 mg/dL), CRP of 8.7 mg/dL (reference range: <0.5 mg/dL), and ESR of 109 mm/hr (reference range: 0-15 mm/hr).

**Figure 1:**
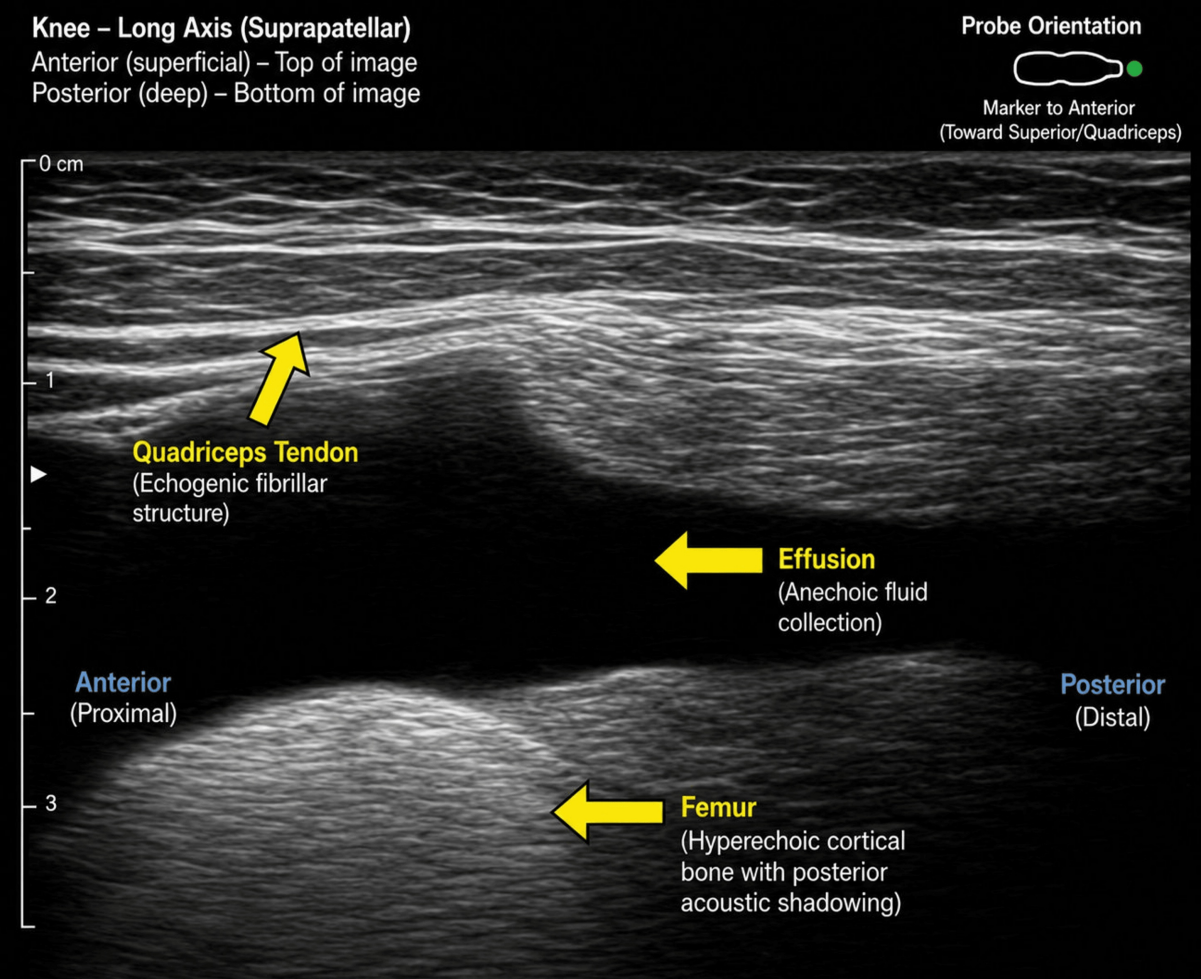
Ultrasound image of the left suprapatellar recess demonstrating anechoic fluid distension consistent with joint effusion.

**Figure 2:**
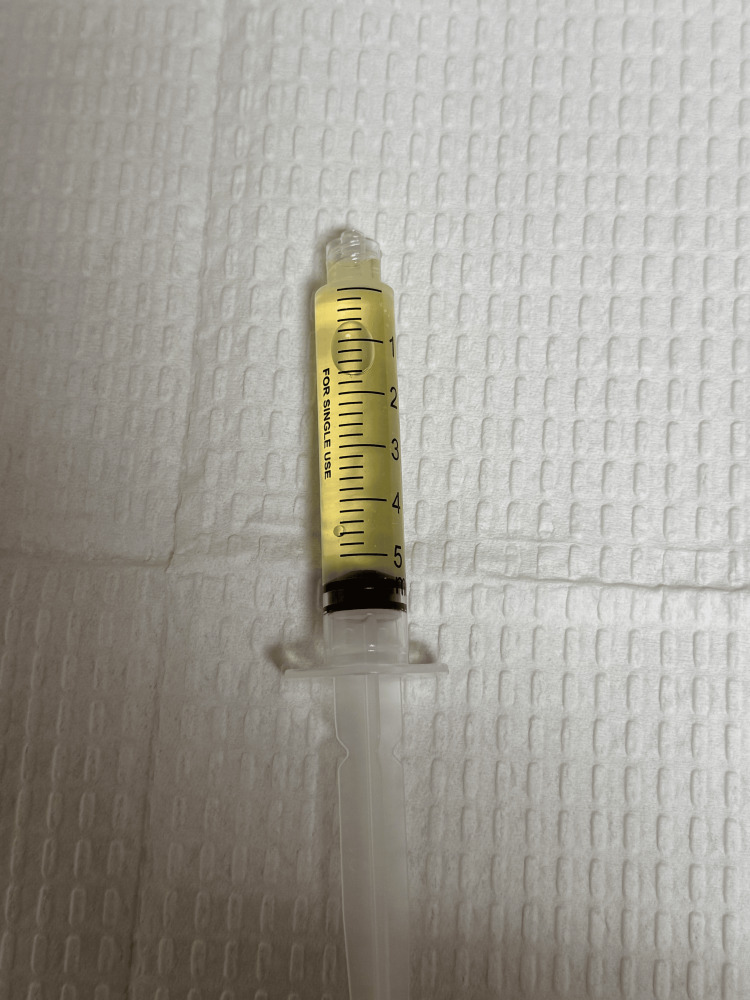
Yellow synovial fluid aspirate obtained during arthrocentesis.

For the procedure, the patient was positioned supine with the leg hanging over the bed and the knee flexed at approximately 30 degrees. The suprapatellar region was cleansed with an antiseptic solution. Ultrasound examination was performed using a Philips Lumify S4-1, 1-4 MHz broadband phased-array transducer, to identify the suprapatellar recess and the optimal entry site for aspiration. As shown in Figure 1, a large effusion was visualized within the suprapatellar recess between the quadriceps tendon and the distal femur. Under real-time ultrasound guidance, the postgraduate year one (PGY-1) internal medicine resident successfully advanced the needle into the suprapatellar recess using an in-plane, lateral-to-medial approach while maintaining continuous visualization of the needle tip. No local anesthetic was administered. On the first attempt by the PGY-1, successful aspiration yielded close to 6 mL of synovial fluid, as seen in Figure 2.

The subsequent synovial fluid analysis is shown in Table 1, compared with alternative etiologies. Removal of the fluid alleviated pain and swelling, with improvement in mobility. Although no crystals were seen on analysis, given his symptoms and score of 8.5 points on the Acute Diagnosis Rule, urate-lowering therapy was started, and the patient was discharged home after two days.

**Table 1:** Synovial fluid analysis compared across key differential diagnoses.

Parameter	Our case	Gout	Septic arthritis	Pseudogout	Osteoarthritis
Synovial fluid color	Yellow	Yellow	Purulent	Yellow	Clear
WBC count (cells/µL)	2,300	2,000-50,000	Usually >50,000	2,000-50,000	<2,000
Gram stain	Negative	Negative	May be positive	Negative	Negative
Culture	No growth	No growth	May show growth	No growth	No growth
Crystals	None	Monosodium urate crystals (needle-shaped, negatively birefringent)	None	Calcium pyrophosphate crystals (rhomboid, positively birefringent)	None

## Discussion

POCUS is a highly versatile tool applicable across multiple clinical settings, making it one of the most impactful advancements in contemporary medical practice.

When applied to joint evaluation, its ability to provide real-time visualization offers significant diagnostic and procedural advantages over landmark-based techniques. In cases of acute monoarticular arthropathy, POCUS is highly useful, especially in the rapid evaluation of septic arthritis, hemarthrosis, and gout. Timely arthrocentesis not only facilitates diagnosis but also enables prompt therapeutic intervention, improving patient outcomes [5].

Ultrasound guidance enhances precision by allowing direct visualization of effusions, needle trajectory, and surrounding anatomical structures. This capability increases the likelihood of successful aspiration with fewer attempts, thereby reducing patient discomfort and procedure-related complications [4,6]. Importantly, studies show that novice operators achieve markedly higher first-attempt success rates with ultrasound localization compared to landmark-only techniques [7].

Our case demonstrates how POCUS can transform procedural performance in early-stage trainees. The PGY-1 resident achieved first-attempt success during their initial knee arthrocentesis, underscoring the dual benefit of accurate diagnosis and immediate skill reinforcement. The visual confirmation provided by POCUS not only guided needle placement but also bolstered the resident’s confidence, a critical factor in procedural learning curves.

While this case demonstrates feasibility in a supervised setting, educational conclusions regarding procedural training require further prospective evaluation. However, we propose that structured integration of ultrasound-guided arthrocentesis into residency training can serve as both a patient safety measure and a powerful educational intervention. By pairing clinical necessity with real-time visual learning, POCUS fosters technical proficiency, accelerates competence, and supports better patient care.

## Conclusions

POCUS-guided arthrocentesis represents a significant advancement over traditional landmark-based techniques. It offers multifaceted benefits to both clinical practice and medical pedagogy. By providing real-time visualization, POCUS significantly improves first-attempt success rates, even for novice operators, while simultaneously accelerating diagnosis and minimizing patient discomfort. Beyond these immediate clinical outcomes, the technology serves as a powerful educational tool. POCUS fosters early procedural confidence and rapid skill acquisition among trainees. Consequently, the structured integration of POCUS training into graduate medical education is more than a technical upgrade. It may represent a cost-effective, high-yield investment that strengthens technical competency and elevates the overall quality of patient care.
